# Differences in iron intake during pregnancy influence in trainability response of male rat offspring

**DOI:** 10.31744/einstein_journal/2020AO5665

**Published:** 2020-11-25

**Authors:** Beatriz Franco, Lucca Antonio Rodrigues Cavallaro, Diego Silva Mota, Natália de Almeida Rodrigues, Fúlvia de Barros Manchado-Gobatto, Rosângela Maria Neves Bezerra, Andrea Maculano Esteves

**Affiliations:** 1 Universidade Estadual de Campinas Faculdade de Educação Física CampinasSP Brazil Faculdade de Educação Física, Universidade Estadual de Campinas, Campinas, SP, Brazil.; 2 Universidade Estadual de Campinas Faculdade de Ciências Aplicadas LimeiraSP Brazil Faculdade de Ciências Aplicadas, Universidade Estadual de Campinas, Limeira, SP, Brazil.

**Keywords:** Lactate minimum, Animal model, Iron supplementation, Iron restriction

## Abstract

**Objective:**

To evaluate if different concentrations of iron in diets during pregnancy would interfere in the aerobic and anaerobic performance of the offspring, observed during 8-week swimming training and measured by lactate minimum test.

**Methods:**

Pregnant rats were divided into four groups with different dietary iron concentrations: standard (40mg/kg), supplementation (100mg/kg), restriction since weaning, and restriction only during pregnancy (4mg/kg). After birth, the offspring were assigned to their respective groups (Standard Offspring, Supplementation Offspring, Restriction Offspring or Restriction Offspring 2). The lactate minimum test was performed at three time points: before starting exercise training, after 4 weeks and after 8 weeks of exercise training.

**Results:**

The Restriction Offspring Group had a significant reduction in the concentration of lactate minimum and in swimming time to exhaustion, after 4 and 8 weeks of training as compared to before training. Therefore, the results showed the Restriction Offspring Group was not able to maintain regularity during training in lactate minimum tests.

**Conclusion:**

Our results suggested the Restriction Offspring Group showed a marked decrease in its performance parameters, which may have occurred due to iron restriction.

## INTRODUCTION

Iron is the functional component of hemoglobin and myoglobin, and an essential nutrient for the efficient transport of oxygen to body tissues. Iron also plays a key role in the electron transport chain, in oxidative phosphorylation within the mitochondria, and red blood cell production.^(^^1^^)^

Iron deficiency (ID) is a common micronutrient deficiency all over the world, and may cause some symptoms, such as fatigue, exhaustion, heart palpitations, and pallor, besides influencing body functions, such as physical performance, thermoregulation, immune response, and neurological functions.^(^^2^^,^^3^^)^

The risk groups for ID include women during pregnancy and postpartum. Many studies have shown that the consequences of ID during a development period, such as gestation, can extend throughout adult life, even with iron replacement. These consequences are behavioral, neuroanatomic, neurochemical, and neurophysiological.^(^^4^^–^^6^^)^ Iron supplementation seems to be adequate to prevent ID in 90% of women during pregnancy and postpartum.^(^^7^^)^

Maternal intake during pregnancy can impact health and cause physical and mental changes throughout the life of the offspring.^(^^8^^,^^9^^)^ In addition, some habits developed during life (such as diet and exercise) contribute to these changes.^(^^10^^)^

However, to date, little is known about the effect of this exposure of mothers to ID or iron supplementation on the physical performance of offspring in adult life.

Some studies have shown that physical exercise can reduce iron-blood concentration, including serum ferritin (sFer), hemoglobin and erythrocytes, and increases the soluble transferrin receptor (sTfR), indicating impaired iron metabolism.^(^^11^^–^^13^^)^

Iron deficiency can attenuate aerobic performance, sincethere is a decrease in hemoglobin levels, oxygen transport in skeletal muscle, maximal oxygen uptake, and ability to withstand submaximal exertion. Moreover, ID negatively interferes in the activity of oxidative enzymes and respiratory proteins. Iron status can affect endurance and energy efficiency.^(^^14^^–^^16^^)^ Thus, an individual who does not have adequate iron levels may show a noticeable drop in his physical performance.^(^^17^^)^

Studies have shown an association between iron status and anaerobic performance.^(^^18^^,^^19^^)^ However, since anaerobic exercise does not require the availability of oxygen, it is difficult to explain these relations. One hypothesis debated is that the process of resynthesis of creatine phosphate used as energy by the adenosine triphosphate (ATP) and creatine phosphate (CP) system is oxygen-dependent.^(^^20^^)^

One way to analyze aerobic and anaerobic parameters in just one assessment session is by means of the lactate minimum test (LMT),^(^^21^^)^ adopted for this purpose in human^(^^22^^)^ and animal models.^(^^23^^)^

## OBJECTIVE

To evaluate if different concentrations of iron in diets during pregnancy would interfere in the aerobic and anaerobic performances of the offspring, observed during 8-week swimming training and measured by lactate minimum test.

## METHODS

### Animals

**Figure 1:**
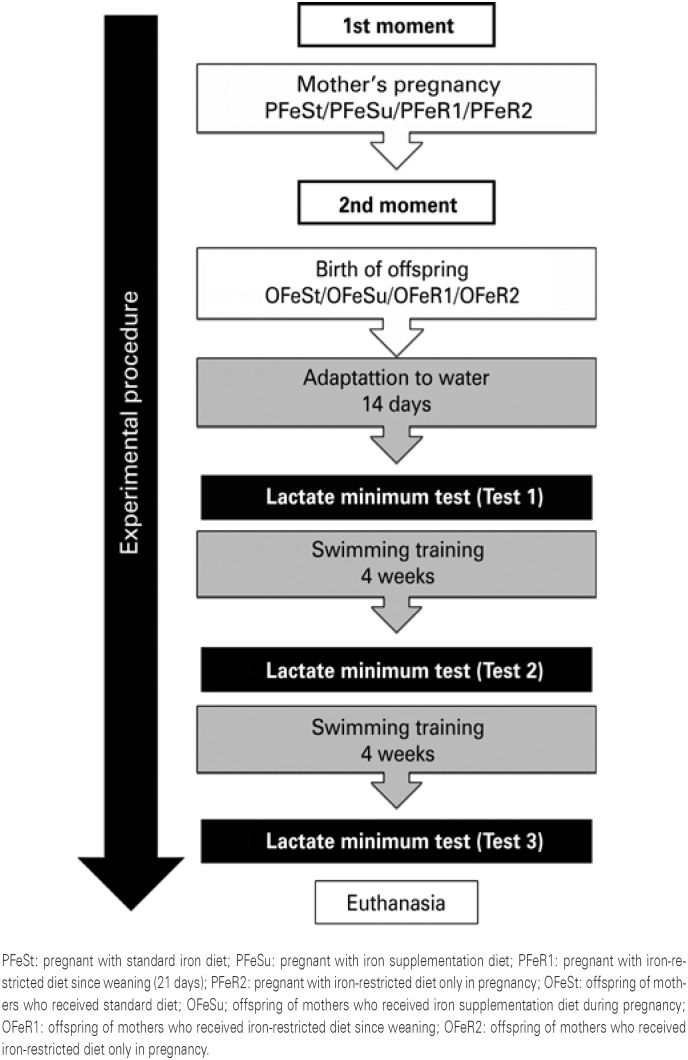
Flowchart showing the groups and exercise

All procedures were approved by the Ethics Committee on the Use of Animals of the Universidade Estadual de Campinas (Unicamp) (number 3876-1). The experiment was carried out in 2 moments ( Figure 1 ): with the mothers and with the offspring.

### First moment

This experiment used 12 female and 3 male Wistar rats, aged 21 days and over, acquired from the Bioethics Center of Centro Multidisciplinar para Investigação Biológica na Área da Ciência em Animais de Laboratório (CEMIB), of Unicamp. Rats were kept transparent cages, four animals per cage, until induction of pregnancy, under controlled conditions of 12-hour light-dark cycle (lights on at 6 a.m.), at a temperature of 23±2°C, with unrestricted access to water and food.

For the induction of pregnancy, two females were placed with one male in each cage. After pregnancy was identified, the mothers were housed in individual cages and randomly distributed into the following groups: pregnant with standard iron diet (PFeSt; n=3); pregnant with iron supplementation diet (PFeSu; n=3); pregnant with iron-restricted diet since weaning - 21 days (PFeR1; n=3); and pregnant with iron-restricted diet only in pregnancy (PFeR2; n=3).

The PFeSt Group remained on the standard iron diet (40mg/kg)^(^^24^^)^ throughout the experiment. The PFeR1 Group received the reduced iron (4mg/kg) diet until the offspring was born. The PFeSu (100mg/kg) and PFeR2 (4mg/kg) groups received the standard diet until the pregnancy was detected, and during the whole pregnancy period, they received the supplementation and restriction diets, respectively. All diets were freely available, and body weight was recorded weekly throughout the experiment. After birth and until weaning the mothers received a standard iron diet (40mg/kg). After 21 days of lactation, the female offspring was separated from the male offspring.

### Second moment

The male offspring were distributed into the groups according to the diet ingested by the mother: offspring of mothers who received a standard diet (OFeSt; n=6); offspring of mothers who received iron supplementation diet during pregnancy (OFeSu; n=8); offspring of mothers who received iron-restricted diet since weaning (OFeR1; n=8); and offspring of mothers who received iron-restricted diet only in pregnancy (OFeR2; n=8).

The offspring was weighed at birth and, after weaning, all groups received standard iron diet. At 70 days of age, the offspring was submitted to procedures of adaptation to a liquid medium progressively for 14 consecutive days. After adaptation, they were evaluated by the LMT for individual determination of anaerobic threshold (AT) and determination of the aerobic condition. The training was performed for 8 weeks, with reevaluations after 4 and 8 weeks of exercise. After the last LMT, the animals were submitted to euthanasia ( Figure 1 ).

### Experimental procedures

#### Iron diet

The diet used to feed the females was produced from purified ingredients, following the composition of the AIN93-G formulations. The values presented in AIN93-G correspond to the needs of the rodent diet in the growth, pregnancy and lactation stages.^(^^25^^)^ The diets for restriction (4mg/kg) and supplementation (100mg/kg) of iron were performed by manipulating the mineral mix, according to the specific percentage for that condition, adjusting the general composition of the mix by the vehicle (sucrose), not altering the caloric density of the diet. The 40mg/kg-diet was used for the Standard Mothers’ Group and Offspring Groups.

Diet samples were sent to the Instituto Adolfo Lutz, São Paulo (SP, Brazil), to verify the iron concentration of each specific diet (standard, restriction, and supplementation).

#### Exercise

The swimming training and the LMT were applied with controlled loads always at the same time (6 p.m.). All procedures were performed in water maintained at 31±1°C and individually, with swimming tanks divided by cylindrical 30cm diameter PVC tubes.

#### Adaptation to water

The animals were submitted to 14 sessions of adaptation to water, being three sessions in water at 15cm of depth. On subsequent days, five sessions of adaptation to free swimming with 120cm of depth, starting with 2 minutes of exercise and increments of 2 minutes daily, until 10 minutes of effort were reached. Next, six specific sessions of adaptation were applied with the mouse swimming and carrying different loads, in percentages of body weight, and time interval varying as a function of the intensity applied. These were only aimed for adaptation to the liquid environment, thermal stress, manipulation, and loads, which remained attached to the animal (back), and were not designed to improve physical condition (training).

#### Lactate minimum test

For determination of aerobic and anaerobic parameters, the LMT protocol described by de Araujo et al.,^(^^26^^)^ was adopted, applied at three-time points during the experimental period: before the training program (animals approximately 84 days old), aiming to identify the individual intensity of AT for training prescription (test 1); after 4 weeks of training (animals approximately 112 days old), to follow the aerobic and anaerobic evolutions and to adjust the training loads (test 2); after 8 weeks of training (animals approximately 140 days old), to identify the training effects on the aerobic and anaerobic parameters analyzed (test 3).

For the LMT and during training, the loads were attached to the animal's back and the percentage of load was calculated for the weight of each animal.

The LMT consisted of a phase of induction of hyperlactatemia, followed by the protocol of incremental loads. The phase of hyperlactatemia was divided into three consecutive parts: 30 seconds effort swimming (load of 13%); 30 seconds of rest interval; and swimming time to exhaustion (TLim) (load of 13%). After the TLim, a passive rest lasting 9 minute (to reach blood lactate peak). After 9 minutes, a protocol of incremental loads was applied to determine the intensity at which the lowest lactatemia was observed, representing the highest intensity of balance between the production and removal of blood lactate. The incremental protocol was performed by swimming with loads of 4%, 4.5%, 5%, 5.5%, 6%, and 7% of the animal weight (lasting 5 minutes for each load) or until exhaustion.

In this way, the lactate minimum concentration was used as an individualized parameter of aerobic capacity and the time to exhaustion during the induction phase as the anaerobic capacity parameter.

Blood samples were drawn from the tip of the animal tail (25μL per collection) through disposable capillary tubes calibrated with heparin.

Subsequently, the extracted blood was stored in Eppendorf tubes with an addition of 50μL of 1% sodium fluoride. Lactate concentrations were determined using the YSI-2300 Yellow Spring lactometer, calibrated and operated according to the manufacturer's specifications.

The value of the balance between production and lactate removal was used to calculate the training load (80% of this value versus body weight). The loads were adjusted weekly.

#### Training program

The individual intensity of exercise corresponded to the product of body weight for 80% of the intensity obtained in the LM, with a volume of 60 minutes per day of swimming, six times a week.^(^^27^^)^ The loads added to the back were readjusted according to the body mass weekly. Physical exercise was performed in the dark period (corresponding to the daytime period of the rats).

### Statistical analysis

The results were analyzed through the Statistica 7.0 software. The mean number of animals per mother was analyzed by analysis of variance (Anova) one-way, followed by Tukey test post-hoc analysis. For analysis of the lactate minimum, TLim (of the LMT) and the weight of offspring, the repeated measure Anova was applied, followed by Tukey test post-hoc analysis. The level of significance was set at p<0.05 and data were expressed as mean±standard error.

## RESULTS

The results did not present statistically significant differences for the mean number of animals per mother (PFeSt: seven animals; PFeSu: ten animals; PFeR1: seven animals, and PFeR2: nine animals). All groups of offspring had a similar pattern of body weight gain throughout the experiment. All groups had a significant increase, relative to themselves, as from 84 days of life.

The results presented here from the LMT were the TLim during the hyperlactatemia phase and contraction of lactate at the moment of balance between production and removal of lactate in the blood (lactate minimum).

**Figure 2:**
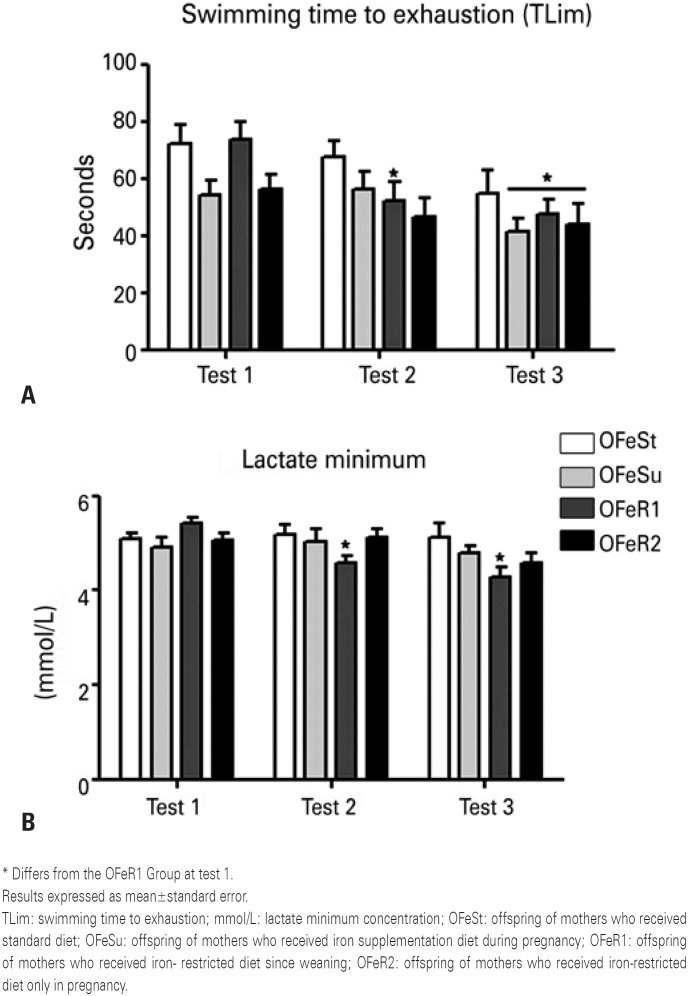
Results of lactate minimum test. (A) swimming time to exhaustion seconds obtained through the lactate minimum test at three time points: baseline (test 1), after 4 weeks of training (test 2) and after 8 weeks of training (test 3). Analysis of variance repeated measures, post Tukey test. Time [F(6.52) = 20,1922; p<0.00001] Group [F(6.52) = 1,7200; p<0.187469] interaction [F(6.52) = 2,2092; p=0.056]. (B) lactate minimum concentration obtained through the lactate minimum test at three time points: baseline (test 1), after 4 weeks of training (test 2) and after 8 weeks of training (test 3). Analysis of variance repeated measures, post Tukey test. Time [F(6.52) = 8,627, p<0.001] group [F(6.52) = 0,889; p=0.459668] interaction [F(6.52) = 4,068; p<0.01]

The OFeR1 Group had a statistically significant reduction of TLim from tests 2 and 3 to test 1, as well as the OFeSu and OFeR2 Groups in test 3 to test 1 of the OFeR1 Group. In addition, a tendency (p=0.06) of limit time reduction was demonstrated for the OFeSu Group in test 3 compared to the OFeSt Group in test 1 ( Figure 2A ). Results showed that the OFeR1 Group had a statistically significant reduction of lactate minimum value in tests 2 and 3 in relation to test 1 ( Figure 2B ).

No statistical differences were found between groups at the time of test 1. The other groups did not show significant alterations in the lactate minimum concentration and TLim after tests 2 and 3 to test 1.

## DISCUSSION

The use of animals in laboratory research helps to investigate stress conditions observed in humans, and enables the monitoring of further changes arising, like iron restriction and exercise.

The study evaluated if different concentrations of iron in diet during pregnancy would interfere in the aerobic and anaerobic performance, measured through the LMT, of rat offspring.

In LMT, our results showed a reduction in aerobic and anaerobic capacity in OFeR1 Group over 8 weeks of training. A possible hypothesis for these changes would be through epigenetics, when there are modifications in the genome through chemical changes that occur in the DNA molecule, which are caused by external factors, such as exercise, diet and stress, and that can be inherited during cellular division.^(^^28^^)^

The literature has shown remarkable results of inheritable epigenetics in an animal models. Some studies have shown that altering the mother's diet affects the metabolism, gene expression of offspring and, consequently, generates metabolic disorders in adult life.^(^^29^^,^^30^^)^

In the study, serum iron and/or gene expression were not performed, however, the diet produced was analyzed; it was found that it presented the specific concentrations for each group.

Even though there were no statistically significant differences in the mean number of animals per mother, the mothers receiving a diet with supplementation had a higher number of pups as compared to other groups. However, it was verified that the weight of the animals throughout the experiment remained similar, not showing changes due to the mother's diet.

The analyses performed for LMT demonstrated the OFeR1 Group showed a significant decrease in lactate values in the first test. The mothers of the OFeR1 Group offspring suffered iron restriction before and during pregnancy, demonstrating a possible relation between diet, epigenetics, and aerobic capacity. However, the OFeR2 Group, in which the mothers received the iron-restricted diet only during pregnancy, did not obtain the same results, suggesting the duration of iron restriction intake influenced the changes.

Studies have shown the reversibility of iron changes in the body depends on the severity and duration of ID.^(^^2^^,^^8^^,^^31^^)^ Also, a study conducted in 2000 with rats fed an iron-deficient diet during pregnancy showed the brain iron levels returned to normal values in 2 weeks of standard iron diet.^(^^21^^)^ Another study with ID during pregnancy showed that iron and monoamine levels in offspring fed standard dietary after weaning were restored.^(^^32^^)^ However, further studies are warranted to understand the different consequences caused by iron restriction for longer or shorter periods.

One of the properties of iron is to be ergogenic, that is, iron can help in the formation of hemoglobin and improve the transport of oxygen in the blood and muscles, and also in the production of energy via oxidative phosphorylation.^(^^33^^)^ The OFeSt and OFeSu Groups did not show significant differences in the values of the tests; therefore, they were able to maintain regularity in the results in all tests of LMT, but without improvement. These results open a space for the hypothesis that a regular iron diet or supplementation during pregnancy can enable the offspring to maintain constancy in their aerobic capacity results, being able to present regularity in their training.

The TLim was established to verify the anaerobic performance of the offspring, in which the OFeR1 Group showed a significant decrease to itself when compared to the first test. The iron restriction of this group did not allow them to progress or maintain their performance throughout the tests. According to some studies, when hemoglobin and iron are not at normal levels it can lead to a significant worsening of anaerobic performance.^(^^19^^,^^34^^)^ Our results showed a reduction in TLim of the OFeSu, OFeR2, and OFeR1 in test 3 compared to OFeR1 in test 1. However, the OFeSt, OFeSu and OFeR2 groups maintained similar results throughout the experiment (test 1, test 2 and, test 3) to themselves.

Research groups around the world are dedicated to understanding the effects of epigenetic changes during pregnancy, and how the offspring's lifestyle habits can interact with these changes. Studies have shown that iron restriction causes epigenetic changes in the hippocampus of mice and mitochondrial proteins.^(^^31^^,^^35^^)^ In this sense, we believe that restriction and supplementation during pregnancy was sufficient to cause changes in iron metabolism, due to the results demonstrated in the offspring.

## CONCLUSION

These results obtained from swimming training of the animals showed that the offspring of mothers who received an iron-restricted diet, before and during pregnancy, showed a marked decrease in their aerobic capacity and anaerobic metabolism indicator through lactate minimum test, while the other groups (offspring of mothers who received standard diet, offspring of mothers who received iron supplementation diet during pregnancy and offspring of mothers who received iron- restricted diet only in pregnancy) had no change in performance over the experiment. However, further studies are required on the subject, since little is known about the consequences of iron restriction on inheritable epigenetics about physical performance.
