# Supplementary material for: Differences in iron intake during pregnancy influence in trainability response of male rat offspring
Source: Einstein (Sao Paulo). 2020 Nov 25;18:eAO5665. doi: 10.31744/einstein_journal/2020AO5665 (PMC7690933; doi:10.31744/einstein_journal/2020AO5665)
Supplement: Translation

# Translation: Diferenças na ingestão de ferro durante a gravidez influenciam na resposta à treinabilidade da prole de ratos machos

**Authors:** Beatriz Franco, Lucca Antonio Rodrigues Cavallaro, Diego Silva Mota, Natália de Almeida Rodrigues, Fúlvia de Barros Manchado-Gobatto, Rosângela Maria Neves Bezerra, Andrea Maculano Esteves

**Affiliations:** 1 CampinasSP Brasil Faculdade de Educação Física, Universidade Estadual de Campinas, Campinas, SP, Brasil.; 2 LimeiraSP Brasil Faculdade de Ciências Aplicadas, Universidade Estadual de Campinas, Limeira, SP, Brasil.

**Keywords:** Lactato mínimo, Modelo animal, Suplementação de ferro, Restrição de ferro

## Abstract

**Objetivo:**

Avaliar parâmetros aeróbios e anaeróbios da prole de ratas alimentadas com diferentes dietas de ferro durante a prenhez, por meio do teste de lactato mínimo.

**Métodos:**

As ratas prenhes foram divididas em quatro grupos com diferentes concentrações de ferro na dieta: padrão (40mg/kg), suplementação (100mg/kg), restrição desde o desmame e restrição apenas durante a gravidez (4mg/kg). Após o nascimento, os filhotes foram designados para seus respectivos grupos (Prole Padrão, Prole Suplementação, Prole Restrição ou Prole Restrição 2). O teste de lactato mínimo foi realizado em três momentos: antes de iniciar o treinamento físico, após 4 semanas e após 8 semanas de treinamento físico.

**Resultados:**

O Grupo Prole Restrição apresentou redução significativa na concentração do lactato mínimo e no tempo de natação até a exaustão, após 4 e 8 semanas de treinamento em relação ao período antes do início do treinamento. Assim, o Grupo Prole Restrição não foi capaz de manter regularidade durante o treinamento nos testes de lactato mínimo.

**Conclusão:**

Os resultados sugerem que o Grupo Prole Restrição apresentou diminuição acentuada nos parâmetros de desempenho, o que pode ter ocorrido devido à restrição de ferro.

## INTRODUÇÃO

O ferro é o componente funcional da hemoglobina e da mioglobina, sendo um nutriente essencial para o transporte eficiente de oxigênio aos tecidos do organismo. Além disso, desempenha papel fundamental na cadeia de transporte de elétrons, na fosforilação oxidativa dentro da mitocôndria e na produção de glóbulos vermelhos.^(^^1^^)^

A deficiência de ferro (DFe) é uma deficiência de micronutrientes comum no mundo todo e pode causar alguns sintomas, como fadiga, exaustão, palpitações e palidez, além de afetar funções orgânicas, como desempenho físico, termorregulação, resposta imune e funções neurológicas.^(^^2^^,^^3^^)^

Os grupos de risco para DFe incluem mulheres durante a gravidez e no pós-parto. Muitos estudos mostraram que as consequências da DFe ocorrida durante um período de desenvolvimento, como a gestação, podem se prolongar ao longo da vida adulta, mesmo com reposição de ferro. Tais consequências são comportamentais, neuroanatômicas, neuroquímicas e neurofisiológicas.^(^^4^^–^^6^^)^A suplementação de ferro parece ser adequada para prevenir DFe em 90% das mulheres durante a gravidez e no pós-parto.^(^^7^^)^

A ingestão materna de ferro durante a gravidez pode impactar na saúde e causar alterações físicas e mentais ao longo da vida da prole.^(^^8^^,^^9^^)^Além disso, há hábitos mantidos ao longo da vida, como alimentação e exercícios, que contribuem para essas alterações.^(^^10^^)^

Porém, até o momento, pouco se sabe do efeito da exposição da mãe à DFe ou da suplementação de ferro no desempenho físico da prole na vida adulta.

Alguns estudos mostraram que o exercício físico pode reduzir a concentração de ferro no sangue, incluindo ferritina sérica (sFer), hemoglobina e eritrócitos, e aumentar a concentração do receptor de transferrina solúvel (sTfR), indicando a presença de um metabolismo de ferro prejudicado.^(^^11^^–^^13^^)^

A DFe pode atenuar o desempenho aeróbio, pois há diminuição dos níveis de hemoglobina, do transporte de oxigênio no músculo esquelético, do consumo máximo de oxigênio e da capacidade de suportar esforços submáximos. Ainda, a DFe interfere de forma negativa na atividade de enzimas oxidativas e proteínas respiratórias. Os níveis de ferro podem afetar a resistência e a eficiência energética.^(^^14^^–^^16^^)^Assim, um indivíduo que não apresenta níveis adequados de ferro pode apresentar queda perceptível em seu desempenho físico.^(^^17^^)^

Há estudos que mostram associação entre os níveis de ferro e o desempenho anaeróbio.^(^^18^^,^^19^^)^No entanto, como o exercício anaeróbio não requer a disponibilidade de oxigênio, é difícil explicar tais relações. Uma hipótese debatida é a de que o processo de ressíntese do fosfato de creatina (usado como energia pelo sistema anaeróbio alático – ATP-CP: adenosine triphosphate - creatine phosphate) é dependente de oxigênio.^(^^20^^)^

Uma forma de analisar os parâmetros aeróbios e anaeróbios em apenas uma sessão de avaliação é por meio do teste de lactato mínimo (TLM),^(^^21^^)^adotado para esse fim em humanos^(^^22^^)^e modelos animais.^(^^23^^)^

## OBJETIVO

Avaliar se diferentes concentrações de ferro na dieta durante a gestação afetam o desempenho aeróbio e anaeróbio da prole, observado durante 8 semanas de treinamento de natação e medido por meio do teste de lactato mínimo.

## MÉTODOS

### Animais

Todos os procedimentos foram aprovados pelo Comitê de Ética no Uso de Animais da Universidade Estadual de Campinas (Unicamp) (nº 3876-1). O experimento foi realizado em dois momentos ( Figura 1 ): com as mães e com a prole.

**Figura 1:** Fluxograma mostrando os grupos e os exercícios

### Primeiro momento

Este experimento utilizou ratos Wistar, sendo 12 fêmeas e três machos, com pelo menos 21 dias de idade, adquiridos no centro de bioética do Centro Multidisciplinar para Investigação Biológica na Área da Ciência em Animais de Laboratório (CEMIB) da Unicamp. Os ratos foram mantidos em gaiolas transparentes, com quatro animais em cada, até a indução da prenhez, sob condições controladas de ciclo claro-escuro de 12 horas (luzes acesas às 6h), a uma temperatura de 23±2°C, com acesso irrestrito à água e à comida.

Para a indução da prenhez, duas fêmeas foram colocadas com um macho em cada gaiola. Após a identificação da prenhez, as mães foram alojadas em gaiolas individuais e distribuídas aleatoriamente nos seguintes grupos: prenhes com dieta padrão de ferro (PFeSt; n=3); prenhes com dieta de suplementação de ferro (PFeSu; n=3); prenhes com dieta com restrição de ferro desde o desmame, de 21 dias (PFeR1; n=3) e prenhes com dieta restrita em ferro apenas na prenhez (PFeR2; n=3).

O Grupo PFeSt manteve a dieta padrão com ferro (40mg/kg)^(^^24^^)^durante todo o experimento. O Grupo PFeR1 recebeu dieta com redução de ferro (4mg/kg) até o nascimento da prole. Os Grupos PFeSu (100mg/kg) e PFeR2 (4mg/kg) receberam a dieta padrão até a detecção da prenhez e, durante todo o período gestacional, receberam as dietas de suplementação e restrição, respectivamente. Todas as dietas estavam disponíveis livremente, e o peso corporal foi registrado a cada semana, durante o experimento. Após o nascimento e até o desmame, as mães receberam uma dieta padrão de ferro (40mg/kg). Após 21 dias de lactação, a prole feminina foi separada da masculina.

### Segundo momento

Os filhotes machos foram distribuídos nos grupos de acordo com a dieta ingerida pela mãe: prole de mães que receberam dieta padrão (OFeSt; n=6); prole de mães que receberam dieta de suplementação de ferro durante a prenhez (OFeSu; n=8); prole de mães que receberam dieta com restrição de ferro desde o desmame (OFeR1; n=8) e prole de mães que receberam dieta com restrição de ferro apenas na prenhez (OFeR2; n=8).

A prole foi pesada ao nascer e, após o desmame, todos os grupos receberam dieta padrão com ferro. Aos 70 dias de idade, os filhotes foram submetidos progressivamente a procedimentos de adaptação a meio líquido por 14 dias consecutivos. Após a adaptação, foram avaliados pelo TLM para determinação individual do limiar anaeróbio (LA) e determinação da condição aeróbia. O treinamento foi realizado por 8 semanas, com reavaliações após 4 e 8 semanas de exercícios. Após o último TLM, os animais foram submetidos à eutanásia ( Figura 1 ).

### Procedimentos experimentais

#### Dieta de ferro

A dieta utilizada na alimentação das fêmeas foi produzida a partir de ingredientes purificados, seguindo a composição das formulações AIN93-G. Os valores apresentados no AIN93-G correspondem às necessidades da dieta de roedores nas fases de crescimento, prenhez e lactação.^(^^25^^)^As dietas de restrição (4mg/kg) e suplementação (100mg/kg) de ferro foram realizadas por manipulação da mistura de minerais, de acordo com o percentual específico para aquela condição, ajustando a composição geral da mistura pelo veículo (sacarose), sem alterar a densidade calórica da dieta. A dieta de 40mg/kg foi usada no grupo de mães padrão e nos grupos de filhotes.

Amostras das dietas foram enviadas ao Instituto Adolfo Lutz, de São Paulo (SP), para verificar a concentração de ferro de cada dieta específica (padrão, restrição e suplementação).

#### Exercício

O treinamento de natação e o TLM foram aplicados com cargas controladas, sempre no mesmo horário (18h). Todos os procedimentos foram realizados em água mantida a 31±1°C e individualmente, com tanques de natação divididos por tubos cilíndricos de PVC de 30cm de diâmetro.

#### Adaptação à água

Os animais foram submetidos a 14 sessões de adaptação à água, sendo três em água com 15cm de profundidade. Nos dias subsequentes, ocorreram cinco sessões de adaptação para natação livre com 120cm de profundidade, iniciando com 2 minutos de exercício e incrementos de 2 minutos diários, até atingir 10 minutos de esforço. Em seguida, foram aplicadas seis sessões específicas de adaptação com diferentes cargas amarradas ao corpo do animal, em percentagens do peso corpóreo, com os tempos variando em função da intensidade aplicada. As sessões se destinavam apenas à adaptação ao meio líquido, ao estresse térmico, à manipulação e às cargas, as quais ficavam presas ao animal (dorso), não objetivando melhorar a condição física (treinamento).

#### Teste mínimo de lactato

Para a determinação dos parâmetros aeróbios e anaeróbios, foi adotado o protocolo TLM descrito por de Araujo et al.,^(^^26^^)^sendo aplicado em três momentos durante o período experimental: antes do programa de treinamento (animais com aproximadamente 84 dias de idade), visando identificar a intensidade individual do LA para a prescrição do treinamento (teste 1); após 4 semanas de treinamento (animais com aproximadamente 112 dias de idade), para acompanhar a evolução aeróbios e anaeróbios e ajustar as cargas de treinamento (teste 2) e após 8 semanas de treinamento (animais com aproximadamente 140 dias de idade), para identificar os efeitos do treinamento sobre os parâmetros aeróbios e anaeróbios analisados (teste 3).

Para o TLM e durante o treinamento, as cargas foram fixadas no dorso do animal, e a percentagens da carga foi calculada para o peso de cada animal.

O TLM consistiu em uma fase de indução de hiperlactatemia, seguida do protocolo de cargas incrementais. A fase de hiperlactatemia foi dividida em três partes consecutivas: 30 segundos de esforço de natação (carga de 13%); 30 segundos de intervalo de descanso e tempo de nado até a exaustão (TLim; carga de 13%). Após o TLim, foi feito repouso passivo, com duração de 9 minutos (para atingir o pico de lactato sanguíneo). Após 9 minutos, foi aplicado um protocolo de cargas incrementais, para determinar a intensidade em que foi observada a menor lactatemia, representando a maior intensidade de equilíbrio entre a produção e a retirada do lactato sanguíneo. O protocolo incremental foi realizado com o animal nadando com cargas de 4%, 4,5%, 5%, 5,5%, 6% e 7% de seu peso (com duração de 5 minutos para cada carga) ou até a exaustão.

Dessa forma, a concentração mínima de lactato foi utilizada como parâmetro individualizado da capacidade aeróbia e o tempo até a exaustão durante a fase de indução, como parâmetro da capacidade anaeróbia.

Foram retiradas amostras de sangue da ponta da cauda do animal (25μL por coleta), por meio de tubos capilares descartáveis e calibrados com heparina.

Posteriormente, o sangue extraído foi armazenado em tubos de Eppendorf com adição de 50μL de fluoreto de sódio a 1%. As concentrações de lactato foram determinadas usando o lactômetro YSI-2300 Yellow Spring, calibrado e operado de acordo com as especificações do fabricante.

O valor do equilíbrio entre a produção e a retirada de lactato foi utilizado para calcular a carga de treinamento (80% desse valor em relação ao peso corpóreo). As cargas foram ajustadas semanalmente.

#### Programa de treinamento

A intensidade individual de exercício correspondeu ao produto do peso corpóreo por 80% da intensidade obtida no LM, com volume de 60 minutos de natação por dia, seis vezes por semana.^(^^27^^)^As cargas somadas ao dorso foram reajustadas de acordo com a massa corpórea semanalmente. O exercício físico foi realizado no período escuro (correspondendo ao período diurno dos ratos).

### Análise estatística

Os resultados foram analisados por meio do programa Statistica 7.0. O número médio de animais por mãe foi avaliado por análise de variância (Anova) simples, seguida de análise post-hoc do teste de Tukey. Para análise do lactato mínimo, TLim (do TLM) e do peso da prole, foi aplicada a Anova com medidas repetidas, seguida do teste de Tukey post-hoc. O nível de significância foi estabelecido como p<0,05, e os dados foram expressos como média±erro padrão.

## RESULTADOS

Os resultados não apresentaram diferenças estatisticamente significativas para o número médio de animais por mãe (PFeSt: sete animais; PFeSu: dez animais; PFeR1: sete animais e PFeR2: nove animais). Todos os grupos de filhotes tiveram um padrão semelhante de ganho de peso corpóreo ao longo do experimento. Todos os grupos tiveram aumento significativo, em relação a eles próprios, a partir dos 84 dias de vida.

Os resultados do TLM aqui apresentados foram o TLim durante a fase de hiperlactatemia e a contração do lactato no momento do equilíbrio, entre a produção e a retirada do lactato no sangue (lactato mínimo).

O Grupo OFeR1 teve redução estatisticamente significativa do TLim dos testes 2 e 3 para o teste 1, assim como os Grupos OFeSu e OFeR2 do teste 3 para o teste 1 do Grupo OFeR1. Além disso, foi demonstrada tendência (p=0,06) de redução do tempo limite para o Grupo OFeSu no teste 3 em relação ao Grupo OFeSt no teste 1 ( Figura 2A ). Os resultados mostraram que o Grupo OFeR1 apresentou redução estatisticamente significativa do valor mínimo de lactato nos testes 2 e 3 em relação ao teste 1 ( Figura 2B ).

**Figura 2:** Resultados do teste de lactato mínimo. (A) medida, em segundos, do tempo de nado até a exaustão obtida por meio do teste de lactato mínimo em três momentos: início/basal (teste 1), após 4 semanas de treinamento (teste 2) e após 8 semanas de treinamento (teste 3). Análise de variância com medidas repetidas, pós-teste de Tukey. Tempo [F (6,52) = 20,1922; p<0,00001] grupo [F (6,52) = 1,7200; p<0,187469] interação [F (6,52) = 2,2092; p=0,056]. (B) concentração do lactato mínimo obtida do teste de lactato mínimo em três momentos: basal (teste 1), após 4 semanas de treinamento (teste 2) e após 8 semanas de treinamento (teste 3). Análise de variância com medidas repetidas, pós-teste de Tukey. Tempo [F (6,52) = 8,627; p<0,001] grupo [F (6,52) = 0,889; p=0,459668] interação [F (6,52) = 4,068; p<0,01]

Não foram encontradas diferenças estatísticas entre os grupos no momento do teste 1. Os demais grupos não apresentaram alterações significativas na concentração mínima de lactato e no TLim, após os testes 2 e 3 para o teste 1.

## DISCUSSÃO

O uso de animais em pesquisas laboratoriais auxilia na investigação das condições de estresse observadas em humanos e possibilita o monitoramento de novas alterações decorrentes disso, como restrição de ferro e exercícios.

O estudo avaliou se diferentes concentrações de ferro na dieta durante a prenhez poderiam interferir no desempenho aeróbio e anaeróbio, medido por meio do TLM, da prole de ratas.

No TLM, nossos resultados mostraram redução na capacidade aeróbia e anaeróbia no Grupo OFeR1 ao longo de 8 semanas de treinamento. Uma possível hipótese para essas alterações seria a epigenética, quando há modificações na expressão do genoma, por meio de alterações químicas que ocorrem na molécula de DNA, que são causadas por fatores externos, como exercícios, dieta alimentar e estresse, e que podem ser herdadas durante a divisão celular.^(^^28^^)^

A literatura mostrou resultados notáveis de epigenética hereditária em modelos animais. Alguns estudos mostraram que uma alteração na dieta da mãe afeta o metabolismo e a expressão gênica da prole e, consequentemente, gera distúrbios metabólicos na vida adulta.^(^^29^^,^^30^^)^

Neste estudo, não foi realizada a expressão gênica e/ou do ferro sérico, mas a dieta produzida foi analisada, e se constatou que apresentava concentrações específicas para cada grupo.

Embora não tenham existido diferenças estatisticamente significativas no número médio de animais por mãe, as mães que receberam dieta com suplementação apresentaram maior número de filhotes em relação aos outros grupos. Porém, verificou-se que o peso dos animais ao longo do experimento permaneceu semelhante, sem apresentar alterações decorrentes da dieta da mãe.

As análises realizadas para o TLM demonstraram que o Grupo OFeR1 apresentou diminuição significativa dos valores de lactato no primeiro teste. As mães da prole do Grupo OFeR1 sofreram restrição de ferro antes e durante a prenhez, demonstrando possível relação entre dieta, epigenética e capacidade aeróbia. Porém, o Grupo OFeR2, no qual as mães receberam dieta restrita em ferro apenas durante a gestação, não obteve os mesmos resultados, sugerindo que o tempo de restrição da ingestão de ferro influenciou nas mudanças.

Estudos têm demonstrado que a reversibilidade das alterações do ferro no organismo depende da gravidade e da duração da DFe.^(^^2^^,^^8^^,^^31^^)^Além disso, um estudo realizado em 2000 com ratas alimentadas com dieta deficiente em ferro durante a prenhez mostrou que os níveis de ferro no cérebro voltaram aos valores normais em 2 semanas de dieta padrão de ferro.^(^^21^^)^Outro estudo com DFe durante a prenhez mostrou que os níveis de ferro e monoamina na prole alimentada com dieta padrão após o desmame foram restaurados.^(^^32^^)^No entanto, mais estudos são necessários para compreender as diferentes consequências da restrição de ferro por períodos mais longos ou mais curtos.

Uma das propriedades do ferro é a de ser ergogênico, ou seja, o ferro pode auxiliar na formação de hemoglobina e melhorar o transporte de oxigênio no sangue e nos músculos, além de ajudar na produção de energia, por meio de fosforilação oxidativa.^(^^33^^)^Os grupos OFeSt e OFeSu não apresentaram diferenças significativas nos valores dos testes, de forma que conseguiram manter a regularidade nos resultados em todos os testes do TLM, porém sem melhora. Esses resultados abrem espaço para a hipótese de que uma dieta regular com ferro ou uma suplementação durante a gestação podem permitir que a prole mantenha constância em seus resultados de capacidade aeróbia, podendo apresentar regularidade em seu treinamento.

O TLim foi estabelecido para verificar o desempenho anaeróbio da prole, e o Grupo OFeR1 apresentou redução significativa em relação a ele próprio quando comparado ao primeiro teste. A restrição de ferro desse grupo não permitiu que ele progredisse ou mantivesse seu desempenho ao longo dos testes. De acordo com alguns estudos, quando a hemoglobina e o ferro não estão em níveis normais, eles podem levar a uma piora significativa do desempenho anaeróbio.^(^^19^^,^^34^^)^Nossos resultados mostraram redução no TLim dos grupos OFeSu, OFeR2 e OFeR1 no teste 3, em comparação com o grupo OFeR1, no teste 1. No entanto, os grupos OFeSt, OFeSu e OFeR2 mantiveram resultados semelhantes ao longo do experimento (teste 1, teste 2 e teste 3) em relação a eles mesmos.

Há grupos de pesquisa em todo o mundo que se dedicam a compreender os efeitos das mudanças epigenéticas durante a gravidez e como os hábitos de vida da prole podem interagir com essas mudanças. Estudos demonstraram que a restrição de ferro causa alterações epigenéticas no hipocampo de camundongos e nas proteínas mitocondriais.^(^^31^^,^^35^^)^Nesse sentido, acreditamos que a restrição e a suplementação durante a gestação foram suficientes para causar alterações no metabolismo do ferro, devido aos resultados demonstrados na prole.

## CONCLUSÃO

Os resultados obtidos no treinamento de natação dos animais mostraram que a prole de mães que receberam dieta restrita em ferro, antes e durante a gestação, apresentou diminuição acentuada em sua capacidade aeróbia e no indicador de metabolismo anaeróbio, por meio do teste de lactato mínimo, ao passo que os demais grupos (prole de mães que receberam dieta padrão, prole de mães que receberam dieta de suplementação de ferro durante a prenhez e prole de mães que receberam dieta com restrição de ferro apenas na gravidez) não tiveram alteração no desempenho durante o experimento. No entanto, mais estudos são necessários sobre o assunto, pois pouco se sabe sobre as consequências da restrição de ferro na epigenética hereditária sobre o desempenho físico.
